# The genome sequence of a cave beetle, *Leptodirus hochenwartii *F.J.Schmidt, 1832

**DOI:** 10.12688/wellcomeopenres.23959.1

**Published:** 2025-03-20

**Authors:** Teo Delić, Wellcome Sanger Institute Tree of Life Management, Samples and Laboratory team, Wellcome Sanger Institute Scientific Operations: Sequencing Operations, Tree of Life Core Informatics collective, Wellcome Sanger Institute Tree of Life Core Informatics team

**Affiliations:** 1 SubBio Laboratory, Department of Biology, University of Ljubljana, Ljubljana, Slovenia

**Keywords:** Leptodirus hochenwartii, genome sequence, chromosomal, Coleoptera, Leptodirini, troglobiont

## Abstract

We present a genome assembly from a male specimen of *Leptodirus hochenwartii* (cave beetle; Arthropoda; Insecta; Coleoptera; Leiodidae). The genome sequence has a total length of 492.36 megabases. Most of the assembly (98.03%) is scaffolded into 14 chromosomal pseudomolecules, including the X and Y sex chromosomes. The mitochondrial genome has also been assembled and is 22.01 kilobases in length.

## Species taxonomy

Eukaryota; Opisthokonta; Metazoa; Eumetazoa; Bilateria; Protostomia; Ecdysozoa; Panarthropoda; Arthropoda; Mandibulata; Pancrustacea; Hexapoda; Insecta; Dicondylia; Pterygota; Neoptera; Endopterygota; Coleoptera; Polyphaga; Staphyliniformia; Staphylinoidea; Leiodidae; Cholevinae; *Leptodirus*; *Leptodirus hochenwartii* F.J.Schmidt, 1832 (NCBI:txid2996356)

## Background

*Leptodirus hochenwartii* Schmidt, 1832 is one of the best-known subterranean species worldwide, as it was the first species described from subterranean habitats ( Polak, 2005). Its discovery was a gamechanger, altering perceptions of these cold and wet environments. Previously, caves were associated with mythical creatures such as dragons, trolls, and fairies. The recognition of *Leptodirus* demonstrated that caves could also support real, living animals.

*Leptodirus* is easily recognised by its narrow ‘neck’—the pronotum—which gave rise to its English name, the narrow-necked cave beetle. Other distinguishing features include its elongated antennae and legs, as well as its heavily inflated hind body (elytra), combined with an amber-reddish colour. This species also lent its name to a tribe of predominantly blind subterranean beetles, Leptodirini (family Leiodidae), which is distributed across the western Palearctic and includes more than 1,200 species ( Moldovan, 2012). While most species in this tribe are only a few millimetres long, *Leptodirus* can reach up to 8 mm in body length, making it one of the largest representatives of its family.

The species is found in the northern part of the Dinaric Karst, a 650 km long mountain range in the Balkans, recognised as a global hotspot for subterranean biodiversity ( Culver & Sket, 2000). *Leptodirus* inhabits caves in north-eastern Italy, Slovenia, and north-western Croatia. To date, only one species has been recognised, with six geographically distinct subspecies ( Pretner, 1970). However, recent analyses suggest that at least some of these subspecies, along with previously unidentified lineages, should be elevated to species level.

Due to its cryptic lifestyle, little is known about its ecology and behaviour. French researchers who kept dozens of individuals in captivity reported that females lay a single egg ( Deleurance-Glacon, 1963). After hatching, larvae survive for five months without feeding before transforming into adult beetles.

*Leptodirus* is one of the few subterranean animals included in the EU’s Natura 2000 conservation network, highlighting its uniqueness. However, since no long-term population monitoring data are available, conservation assessments should be interpreted with caution. This is particularly important given that the species’ current 300 km range could fragment into a series of smaller distributions if some subspecies are reclassified as distinct species ( Bregović *et al.*, 2019). Understanding its genome would not only aid conservation efforts but also provide a foundation for studying the phylogeny of the entire *Leptodirini* tribe.

## Genome sequence report

### Sequencing data

**Figure 1.**
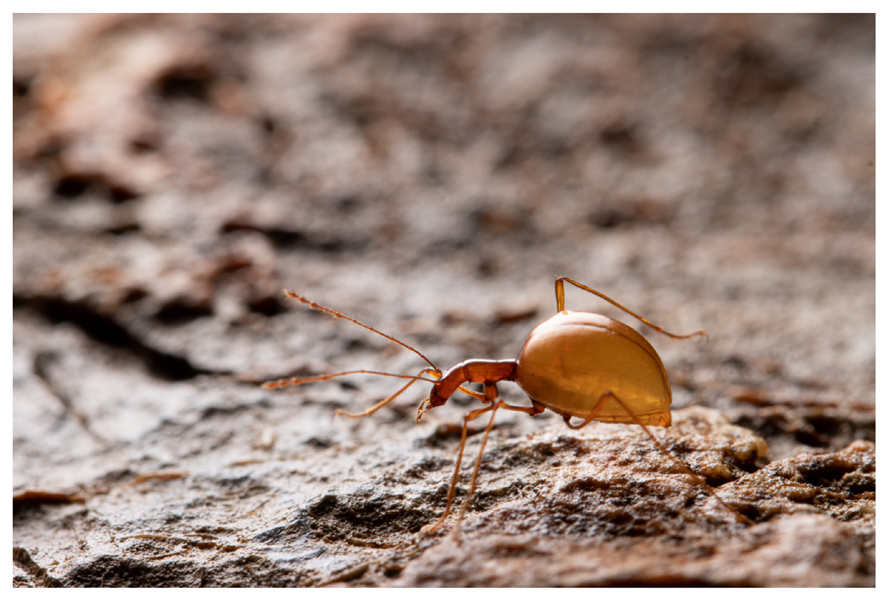
Photograph of the *Leptodirus hochenwartii* (icLepHoch12) specimen used for genome sequencing.

The genome of a male specimen of *Leptodirus hochenwartii* ( Figure 1) was sequenced using Pacific Biosciences single-molecule HiFi long reads, generating 21.65 Gb (gigabases) from 1.86 million reads. Based on the estimated genome size in GenomeScope, the sequencing data provided approximately 47.0x coverage of the genome. Chromosome conformation Hi-C sequencing produced 133.06 Gb from 881.18 million reads. Table 1 summarises the specimen and sequencing information.

**Table 1.** Specimen and sequencing data for *Leptodirus hochenwartii*.

Project information			
**Study title**	Leptodirus hochenwartii (narrow-necked blind cave beetle)		
**Umbrella BioProject**	PRJEB56110		
**Species**	*Leptodirus hochenwartii*		
**BioSpecimen**	SAMEA11319724		
**NCBI taxonomy ID**	2996356		
Specimen information			
**Technology**	**ToLID**	**BioSample accession**	**Organism part**
**PacBio long read sequencing**	icLepHoch12	SAMEA11319735	whole organism
**Hi-C sequencing**	icLepHoch12	SAMEA11319735	whole organism
**RNA sequencing**	icLepHoch3	SAMEA11319726	whole organism
Sequencing information			
**Platform**	**Run accession**	**Read count**	**Base count (Gb)**
**Hi-C Illumina NovaSeq 6000**	ERR10297840	8.81e+08	133.06
**PacBio Sequel IIe**	ERR10287558	1.86e+06	21.65
**RNA Illumina NovaSeq 6000**	ERR10908605	7.05e+07	10.64

### Assembly statistics

The primary haplotype was assembled, and contigs corresponding to an alternate haplotype were also deposited in INSDC databases. The assembly was improved by manual curation, which corrected 274 misjoins or missing joins and removed 15 haplotypic duplications. These interventions reduced the total assembly length by 1.41%, decreased the scaffold count by 77.74%, and increased the scaffold N50 by 186.13%. The final assembly has a total length of 492.36 Mb in 74 scaffolds, and a scaffold N50 of 37.27 Mb ( Table 2).

**Table 2.** Genome assembly data for *Leptodirus hochenwartii*.

Genome assembly		
Assembly name	icLepHoch12.1	
Assembly accession	GCA_947310635.1	
*Alternate haplotype accession*	*GCA_947310685.1*	
Assembly level for primary assembly	chromosome	
Span (Mb)	492.36	
Number of contigs	408	
Number of scaffolds	74	
Longest scaffold (Mb)	51.37	
Assembly metric	Measure	*Benchmark*
Contig N50 length	3.37 Mb	*≥ 1 Mb*
Scaffold N50 length	37.27 Mb	*= chromosome N50*
Consensus quality (QV)	65.3 (combined)	*≥ 40*
*k*-mer completeness	Primary: 87.43%; alternate: 82.49%; combined: 96.74%	*≥ 95%*
BUSCO *	C:97.4%[S:96.8%,D:0.6%], F:1.4%,M:1.2%,n:2,124	*S > 90%; D < 5%*
Percentage of assembly mapped to chromosomes	97.38%	*≥ 90%*
Sex chromosomes	X and Y	*localised homologous pairs*
Organelles	Mitochondrial genome: 22.01 kb	*complete single alleles*

* BUSCO scores based on the endopterygota_odb10 BUSCO set using version 5.3.2. C = complete [S = single copy, D = duplicated], F = fragmented, M = missing, n = number of orthologues in comparison.

**Figure 2.**
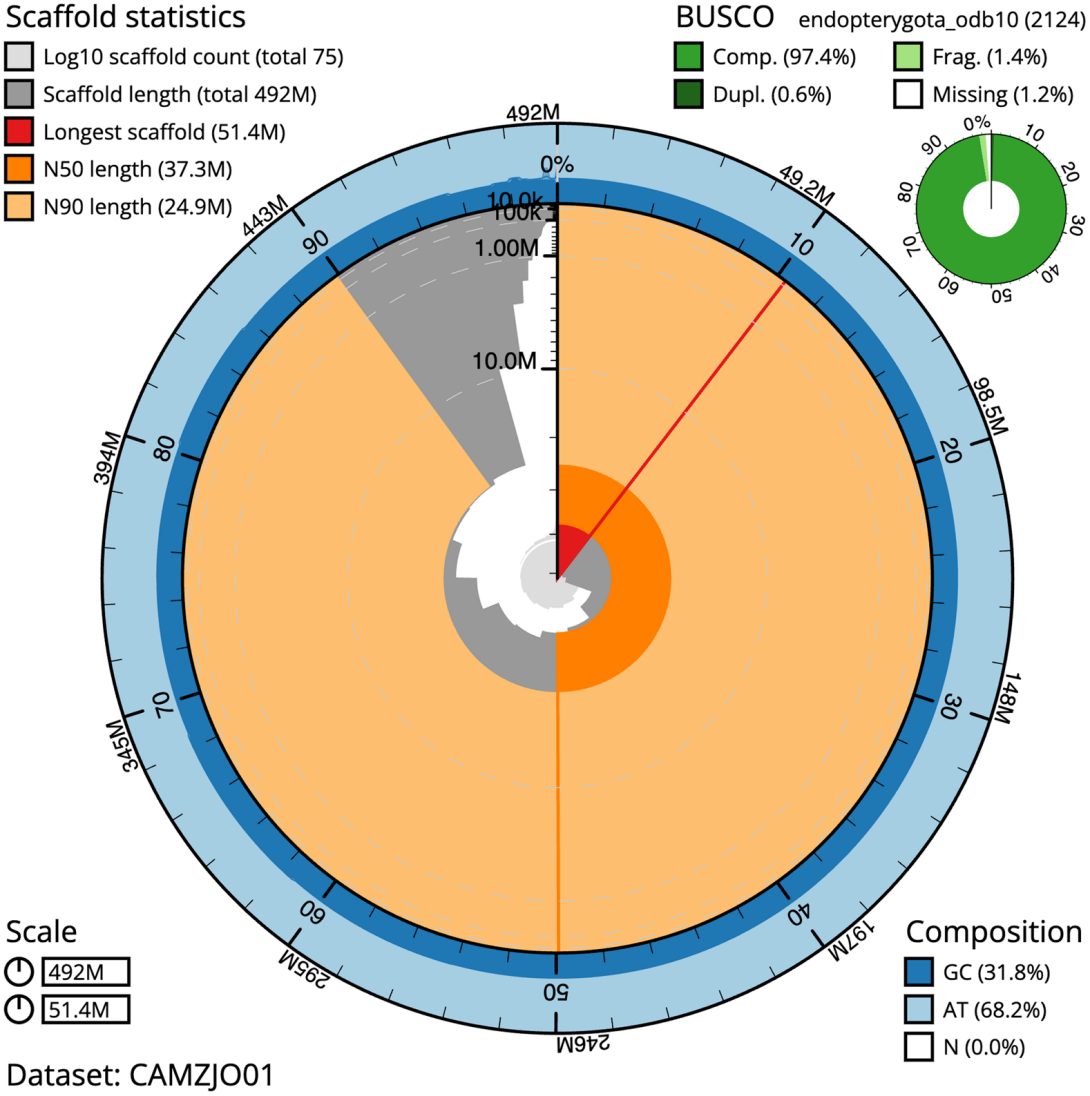
Genome assembly of *Leptodirus hochenwartii*, icLepHoch12.1: metrics. The BlobToolKit snail plot provides an overview of assembly metrics and BUSCO gene completeness. The circumference represents the length of the whole genome sequence, and the main plot is divided into 1,000 bins around the circumference. The outermost blue tracks display the distribution of GC, AT, and N percentages across the bins. Scaffolds are arranged clockwise from longest to shortest and are depicted in dark grey. The longest scaffold is indicated by the red arc, and the deeper orange and pale orange arcs represent the N50 and N90 lengths. A light grey spiral at the centre shows the cumulative scaffold count on a logarithmic scale. A summary of complete, fragmented, duplicated, and missing BUSCO genes in the endopterygota_odb10 set is presented at the top right. An interactive version of this figure is available at https://blobtoolkit.genomehubs.org/view/Leptodirus%20hochenwartii/dataset/CAMZJO01/snail.

**Figure 3.**
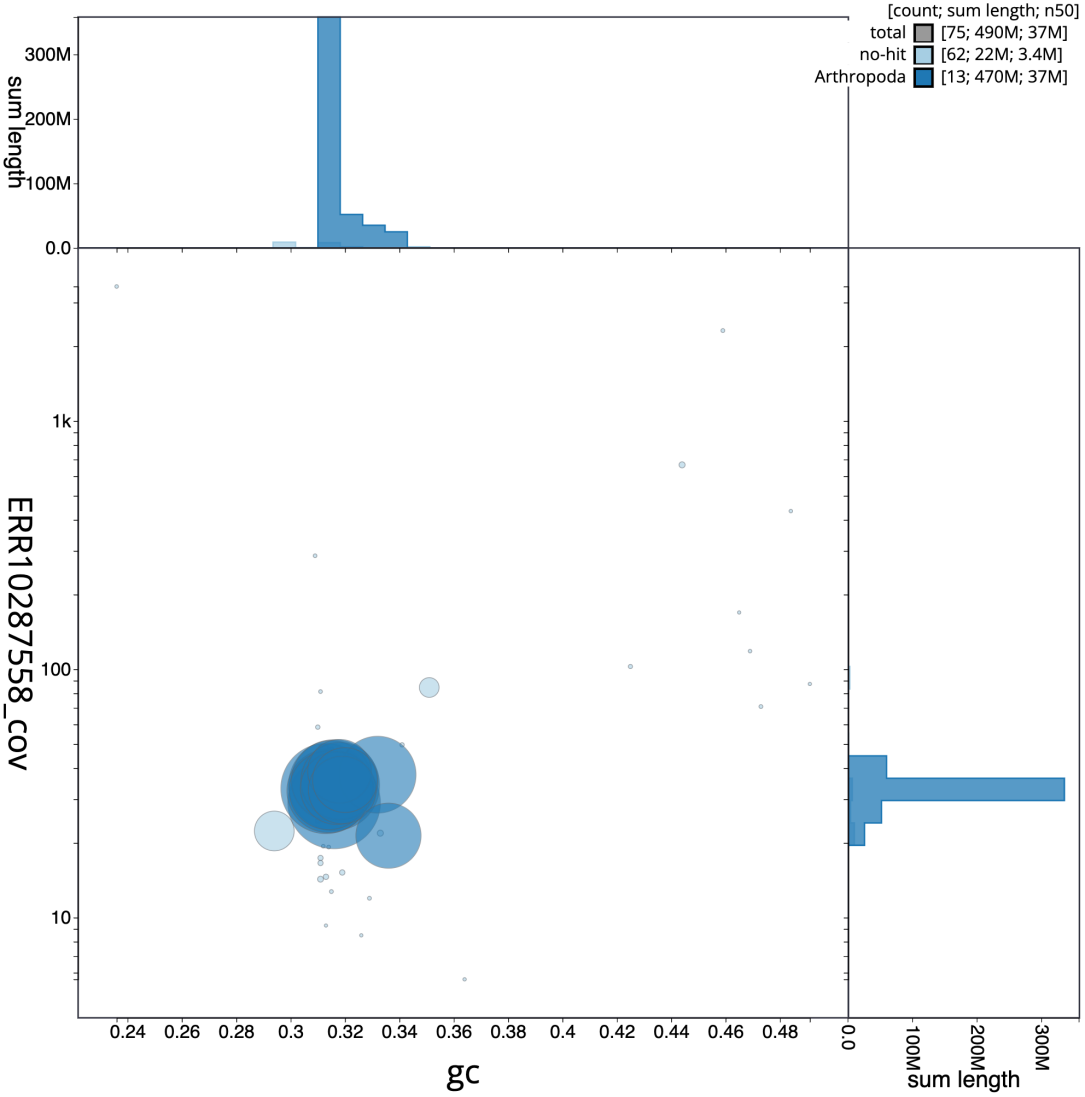
Genome assembly of *Leptodirus hochenwartii*, icLepHoch12.1: BlobToolKit GC-coverage plot. Blob plot showing sequence coverage (vertical axis) and GC content (horizontal axis). The circles represent scaffolds, with the size proportional to scaffold length and the colour representing phylum membership. The histograms along the axes display the total length of sequences distributed across different levels of coverage and GC content. An interactive version of this figure is available at https://blobtoolkit.genomehubs.org/view/Leptodirus%20hochenwartii/dataset/CAMZJO01/blob.

**Figure 4.**
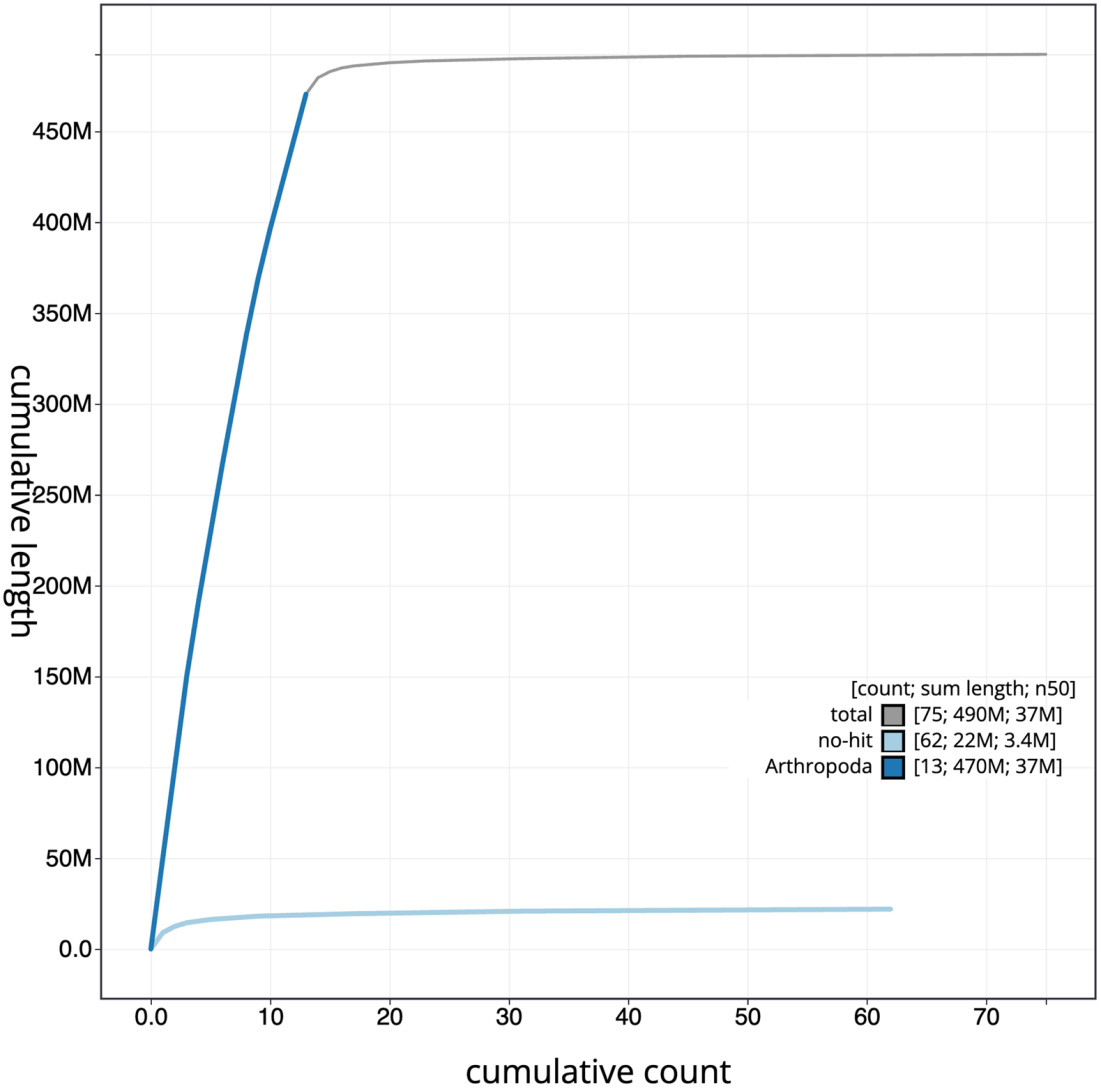
Genome assembly of *Leptodirus hochenwartii,* icLepHoch12.1: BlobToolKit cumulative sequence plot. The grey line shows cumulative length for all scaffolds. Coloured lines show cumulative lengths of scaffolds assigned to each phylum using the buscogenes taxrule. An interactive version of this figure is available at https://blobtoolkit.genomehubs.org/view/Leptodirus%20hochenwartii/dataset/CAMZJO01/cumulative.

The snail plot in Figure 2 provides a summary of the assembly statistics, indicating the distribution of scaffold lengths and other assembly metrics. Figure 3 shows the distribution of scaffolds by GC proportion and coverage. Figure 4 presents a cumulative assembly plot, with separate curves representing different scaffold subsets assigned to various phyla, illustrating the completeness of the assembly.

**Figure 5.**
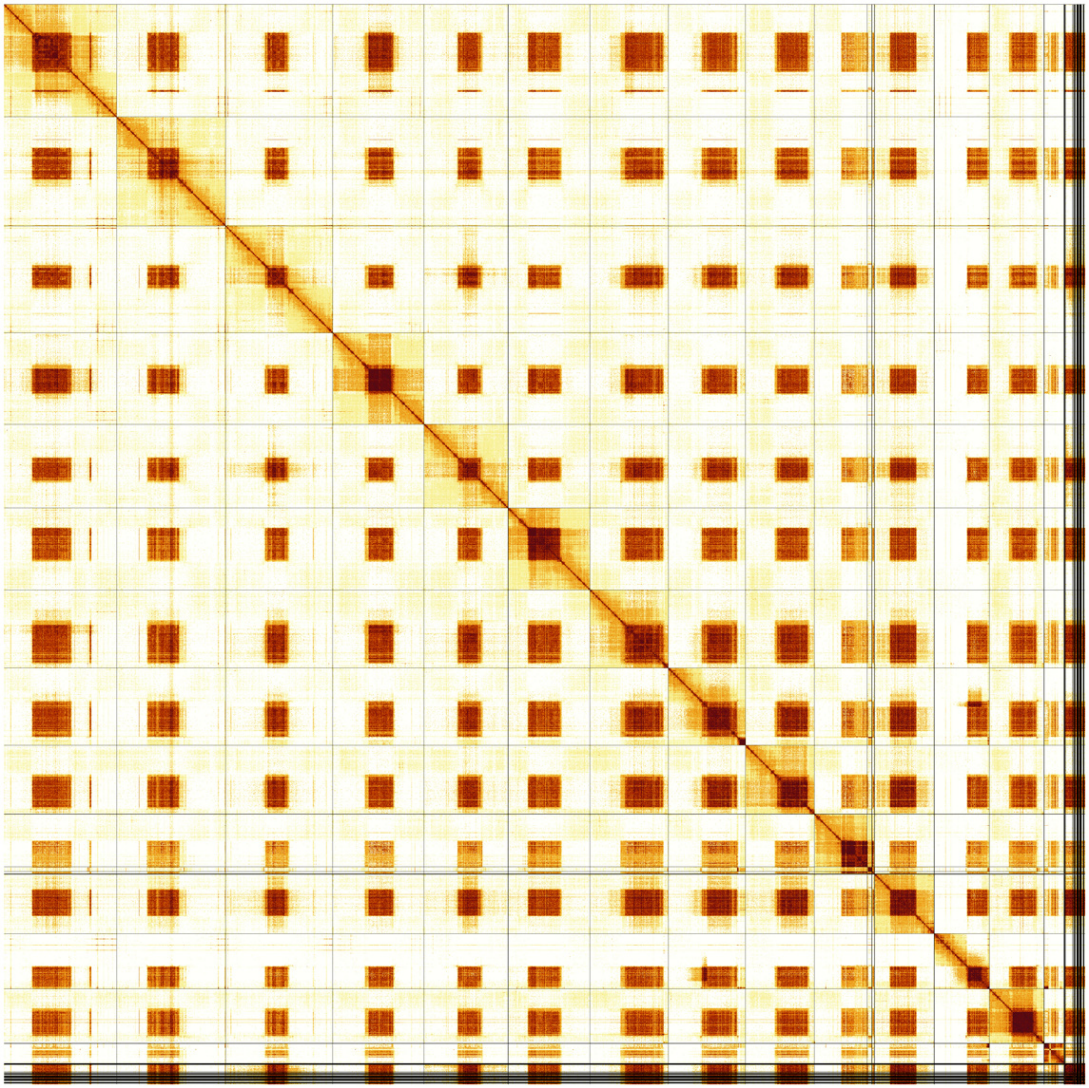
Genome assembly of *Leptodirus hochenwartii*: Hi-C contact map of the icLepHoch12.1 assembly, produced in PretextView. Chromosomes are shown in order of size from left to right and top to bottom.

Most of the assembly sequence (97.38%) was assigned to 14 chromosomal-level scaffolds, representing 12 autosomes and the X and Y sex chromosomes. These chromosome-level scaffolds, confirmed by Hi-C data, are named according to size ( Figure 5; Table 3). Due to the repetitive nature of the sequence the order and orientation of scaffolds in the following regions are unsure: Chromosome 1 ~14.10–29.54 Mb, Chromosome 2 ~19.21–24.26 Mb, Chromosome 3 ~18.39–26.05, Chromosome 4 ~16.28–26.44 Mb, Chromosome 5 ~17.23-24.40 Mb, Chromosome 6 ~9.90–22.38 Mb, Chromosome 7 ~16.55–33.23 Mb, Chromosome 8 ~17.79–35.15 Mb, Chromosome 9 ~16.21–27.67 Mb, Chromosome 10 ~12.84–24.02 Mb, Chromosome 11 ~8.29–18.51 Mb, Chromosome 12 ~11.79–15.64 Mb.

**Table 3.** Chromosomal pseudomolecules in the genome assembly of *Leptodirus hochenwartii*, icLepHoch12.

INSDC accession	Name	Length (Mb)	GC%
OX371176.1	1	51.37	31.5
OX371177.1	2	49.64	31.5
OX371178.1	3	48.78	31.5
OX371179.1	4	41.6	31.5
OX371180.1	5	38.24	31.5
OX371181.1	6	37.27	31.5
OX371182.1	7	35.63	32
OX371183.1	8	35.17	33
OX371184.1	9	31.49	31.5
OX371185.1	10	24.04	32
OX371186.1	11	27.14	32
OX371188.1	12	24.91	32
OX371187.1	X	25.14	33.5
OX371189.1	Y	9.03	29.5
OX371190.1	MT	0.02	24.5

The mitochondrial genome was also assembled. This sequence is included as a contig in the multifasta file of the genome submission and as a standalone record in GenBank.

### Assembly quality metrics

The estimated Quality Value (QV) and *k*-mer completeness metrics, along with BUSCO completeness scores, were calculated for each haplotype and the combined assembly. The QV reflects the base-level accuracy of the assembly, while *k*-mer completeness indicates the proportion of expected *k*-mers identified in the assembly. BUSCO scores provide a measure of completeness based on benchmarking universal single-copy orthologues.

The combined primary and alternate assemblies achieve an estimated QV of 65.3 (an error rate of less than 1 per 1 million bases). The *k*-mer completeness for the primary haplotype is 87.43%, and for the alternate haplotype 82.49%, while the combined primary and alternate assemblies achieve a *k*-mer completeness of 96.74%. BUSCO (v 5.3.2) analysis recovered 97.4% (single = 96.8%, duplicated = 0.6%) of the endopterygota_odb10 reference set ( *n* = 2,124).

Table 2 provides assembly metric benchmarks adapted from Rhie *et al.* (2021) and the Earth BioGenome Project Report on Assembly Standards September 2024. The assembly achieves the EBP reference standard of **6.C.Q65**.

## Methods

### Sample acquisition

An adult male *Leptodirus hochenwartii* (specimen ID SAN0001791, ToLID icLepHoch12) was collected from Cave Mačkovica, Laze, Logatec (latitude 45.8589, longitude 14.2707) on 2021-07-22. This specimen was used for PacBio HiFi DNA sequencing. A second specimen (specimen ID SAN0001782, ToLID icLepHoch3) was used for RNA sequencing. The specimens were collected by Teo Delić and Danaja Zagmajster Delić (Biotechnical Faculty, University of Ljubljana) and identified by Teo Delić.

### Nucleic acid extraction

The workflow for high molecular weight (HMW) DNA extraction at the Wellcome Sanger Institute (WSI) Tree of Life Core Laboratory includes a sequence of procedures: sample preparation and homogenisation, DNA extraction, fragmentation and purification. Detailed protocols are available on protocols.io ( Denton *et al.*, 2023b).

The icLepHoch12 sample was prepared for DNA extraction by weighing and dissecting it on dry ice ( Jay *et al.*, 2023). Tissue from the whole organism was homogenised using a PowerMasher II tissue disruptor ( Denton *et al.*, 2023a). HMW DNA was extracted using the Automated MagAttract v1 protocol ( Sheerin *et al.*, 2023). DNA was sheared into an average fragment size of 12–20 kb in a Megaruptor 3 system ( Todorovic *et al.*, 2023). Sheared DNA was purified by solid-phase reversible immobilisation, using AMPure PB beads to eliminate shorter fragments and concentrate the DNA ( Strickland *et al.*, 2023). The concentration of the sheared and purified DNA was assessed using a Nanodrop spectrophotometer and a Qubit Fluorometer using the Qubit dsDNA High Sensitivity Assay kit. The fragment size distribution was evaluated by running the sample on the FemtoPulse system.

RNA was extracted from whole organism tissue of icLepHoch3 in the Tree of Life Laboratory at the WSI using the RNA Extraction: Automated MagMax™ *mir*Vana protocol ( do Amaral *et al.*, 2023). The RNA concentration was assessed using a Nanodrop spectrophotometer and a Qubit Fluorometer using the Qubit RNA Broad-Range Assay kit. Analysis of the integrity of the RNA was done using the Agilent RNA 6000 Pico Kit and Eukaryotic Total RNA assay.

### Hi-C sample preparation

Tissue from the icLepHoch12 sample was processed for Hi-C sequencing at the WSI Scientific Operations core, using the Arima-HiC v2 kit. In brief, 20–50 mg of frozen tissue (stored at –80 °C) was fixed, and the DNA crosslinked using a TC buffer with 22% formaldehyde concentration. After crosslinking, the tissue was homogenised using the Diagnocine Power Masher-II and BioMasher-II tubes and pestles. Following the Arima-HiC v2 kit manufacturer's instructions, crosslinked DNA was digested using a restriction enzyme master mix. The 5’-overhangs were filled in and labelled with biotinylated nucleotides and proximally ligated. An overnight incubation was carried out for enzymes to digest remaining proteins and for crosslinks to reverse. A clean up was performed with SPRIselect beads prior to library preparation. Additionally, the biotinylation percentage was estimated using the Qubit Fluorometer v4.0 (Thermo Fisher Scientific) and Qubit HS Assay Kit and Arima-HiC v2 QC beads.

### Library preparation and sequencing

Library preparation and sequencing were performed at the WSI Scientific Operations core.

***PacBio HiFi***

Libraries were prepared using the PacBio Express Template Preparation Kit v2.0 (Pacific Biosciences, California, USA) as per the manufacturer's instructions. The kit includes the reagents required for removal of single-strand overhangs, DNA damage repair, end repair/A-tailing, adapter ligation, and nuclease treatment. Library preparation also included a library purification step using AMPure PB beads (Pacific Biosciences, California, USA) and size selection step to remove templates shorter than 3 kb using AMPure PB modified SPRI. DNA concentration was quantified using the Qubit Fluorometer v2.0 (Thermo Fisher Scientific) and Qubit HS Assay Kit and the final library fragment size analysis was carried out using the Agilent Femto Pulse Automated Pulsed Field CE Instrument (Agilent Technologies).

Samples were sequenced using the Sequel IIe system (Pacific Biosciences, California, USA). The concentration of the library loaded onto the Sequel IIe was in the range 40–135 pM. The SMRT link software, a PacBio web-based end-to-end workflow manager, was used to set-up and monitor the run, as well as perform primary and secondary analysis of the data upon completion.

***Hi-C***

For Hi-C library preparation, DNA was fragmented using the Covaris E220 sonicator (Covaris) and size selected using SPRISelect beads to 400 to 600 bp. The DNA was then enriched using the Arima-HiC v2 kit Enrichment beads. Using the NEBNext Ultra II DNA Library Prep Kit (New England Biolabs) for end repair, A-tailing, and adapter ligation. This uses a custom protocol which resembles the standard NEBNext Ultra II DNA Library Prep protocol but where library preparation occurs while DNA is bound to the Enrichment beads. For library amplification, 10 to 16 PCR cycles were required, determined by the sample biotinylation percentage. The Hi-C sequencing was performed using paired-end sequencing with a read length of 150 bp on an Illumina NovaSeq 6000 instrument.

***RNA***

Poly(A) RNA-Seq libraries were constructed using the NEB Ultra II RNA Library Prep kit, following the manufacturer’s instructions. RNA sequencing was performed on the Illumina NovaSeq 6000 instrument.

### Genome assembly, curation and evaluation

***Assembly***

Prior to assembly of the PacBio HiFi reads, a database of *k*-mer counts ( *k* = 31) was generated from the filtered reads using FastK. GenomeScope2 ( Ranallo-Benavidez *et al.*, 2020) was used to analyse the *k*-mer frequency distributions, providing estimates of genome size, heterozygosity, and repeat content.

The HiFi reads were first assembled using Hifiasm ( Cheng *et al.*, 2021) with the --primary option. Haplotypic duplications were identified and removed using purge_dups ( Guan *et al.*, 2020). The Hi-C reads were mapped to the primary contigs using bwa-mem2 ( Vasimuddin *et al.*, 2019). The contigs were further scaffolded using the provided Hi-C data ( Rao *et al.*, 2014) in YaHS ( Zhou *et al.*, 2023) using the --break option for handling potential misassemblies. The scaffolded assemblies were evaluated using Gfastats ( Formenti *et al.*, 2022), BUSCO ( Manni *et al.*, 2021) and MERQURY.FK ( Rhie *et al.*, 2020).

The mitochondrial genome was assembled using MitoHiFi ( Uliano-Silva *et al.*, 2023), which runs MitoFinder ( Allio *et al.*, 2020) and uses these annotations to select the final mitochondrial contig and to ensure the general quality of the sequence.

***Assembly curation***

The assembly was decontaminated using the Assembly Screen for Cobionts and Contaminants (ASCC) pipeline (article in preparation). Manual curation was primarily conducted using PretextView ( Harry, 2022), with additional insights provided by JBrowse2 ( Diesh *et al.*, 2023) and HiGlass ( Kerpedjiev *et al.*, 2018). Scaffolds were visually inspected and corrected as described by Howe *et al*. (2021). Any identified contamination, missed joins, and mis-joins were corrected, and duplicate sequences were tagged and removed. The curation process is documented at https://gitlab.com/wtsi-grit/rapid-curation (article in preparation).

***Assembly quality assessment***

The Merqury.FK tool ( Rhie *et al.*, 2020), run in a Singularity container ( Kurtzer *et al.*, 2017), was used to evaluate *k*-mer completeness and assembly quality for the primary and alternate haplotypes using the *k*-mer databases ( *k* = 31) that were computed prior to genome assembly. The analysis outputs included assembly QV scores and completeness statistics.

The genome was also analysed within the BlobToolKit environment ( Challis *et al.*, 2020) and BUSCO scores ( Manni *et al.*, 2021) were calculated.

Table 4 contains a list of relevant software tool versions and sources.

**Table 4.** Software tools: versions and sources.

Software tool	Version	Source
BLAST	2.12.0	ftp://ftp.ncbi.nlm.nih.gov/blast/executables/blast+/
BlobToolKit pipeline release	4.1.6	https://github.com/blobtoolkit/blobtoolkit
BUSCO	5.3.2	https://gitlab.com/ezlab/busco
bwa-mem2	2.2.1	https://github.com/bwa-mem2/bwa-mem2
DIAMOND	2.0.15	https://github.com/bbuchfink/diamond
fasta_windows	0.2.4	https://github.com/tolkit/fasta_windows
FastK	666652151335353eef2fcd58880bcef5bc2928e1	https://github.com/thegenemyers/FASTK
Gfastats	1.3.6	https://github.com/vgl-hub/gfastats
Hifiasm	0.16.1-r375	https://github.com/chhylp123/hifiasm
HiGlass	44086069ee7d4d3f6f3f0012569789ec138f42b84 aa44357826c0b6753eb28de	https://github.com/higlass/higlass
MerquryFK	d00d98157618f4e8d1a9190026b19b471055b22e	https://github.com/thegenemyers/MERQURY.FK
Minimap2	2.24-r1122	https://github.com/lh3/minimap2
PretextView	0.2.5	https://github.com/sanger-tol/PretextView
purge_dups	1.2.3	https://github.com/dfguan/purge_dups
sanger-tol/ascc	-	https://github.com/sanger-tol/ascc
Seqtk	1.3	https://github.com/lh3/seqtk
Singularity	3.9.0	https://github.com/sylabs/singularity
YaHS	yahs-1.1.91eebc2	https://github.com/c-zhou/yahs

### Wellcome Sanger Institute – Legal and Governance

The materials that have contributed to this genome note have been supplied by a Tree of Life collaborator.

The Wellcome Sanger Institute employs a process whereby due diligence is carried out proportionate to the nature of the materials themselves, and the circumstances under which they have been/are to be collected and provided for use. The purpose of this is to address and mitigate any potential legal and/or ethical implications of receipt and use of the materials as part of the research project, and to ensure that in doing so we align with best practice wherever possible.

The overarching areas of consideration are:

•    Ethical review of provenance and sourcing of the material

•    Legality of collection, transfer and use (national and international)

Each transfer of samples is undertaken according to a Research Collaboration Agreement or Material Transfer Agreement entered into by the Tree of Life collaborator, Genome Research Limited (operating as the Wellcome Sanger Institute) and in some circumstances other Tree of Life collaborators.

## Data Availability

European Nucleotide Archive: Leptodirus hochenwartii (narrow-necked blind cave beetle). Accession number PRJEB56110; https://identifiers.org/ena.embl/PRJEB56110. The genome sequence is released openly for reuse. The *Leptodirus hochenwartii* genome sequencing initiative is part of European Reference Genome Atlas Pilot Project ( https://www.erga-biodiversity.eu/pilot-project). All raw sequence data and the assembly have been deposited in INSDC databases. The genome will be annotated using available RNA-Seq data and presented through the Ensembl pipeline at the European Bioinformatics Institute. Raw data and assembly accession identifiers are reported in Table 1 and Table 2.

## Author information

Members of the Wellcome Sanger Institute Tree of Life Management, Samples and Laboratory team are listed here: https://doi.org/10.5281/zenodo.12162482.

Members of Wellcome Sanger Institute Scientific Operations: Sequencing Operations are listed here: https://doi.org/10.5281/zenodo.12165051.

Members of the Wellcome Sanger Institute Tree of Life Core Informatics team are listed here: https://doi.org/10.5281/zenodo.12160324.

Members of the Tree of Life Core Informatics collective are listed here: https://doi.org/10.5281/zenodo.12205391.
