# Supplementary material for: The genome sequence of a cave beetle, Leptodirus hochenwartii F.J.Schmidt, 1832
Source: Wellcome Open Res. 2025 Mar 20;10:159. [Version 1] doi: 10.12688/wellcomeopenres.23959.1 (PMC11992515; doi:10.12688/wellcomeopenres.23959.1)
Supplement: Peer review history

# Reviewer report: Reviewer response for version 1

**DOI:** 10.21956/wellcomeopenres.26435.r121998

**Published:** 2025-04-11

**Authors:** Hume Douglas

**Affiliations:** 1 Agriculture and Agri-Food Canada, Ottawa, Canada

Do you need to mention any permits or authority required to obtain the specimen? Is this a protected species in Slovenia?

 What literature was used to identify the specimen? It was a live specimen, so what literature would a reader use to identify another specimen to species level?

 This will be an important resource for understanding cave beetles and Leiodidae.

Are sufficient details of methods and materials provided to allow replication by others?

Yes

Is the rationale for creating the dataset(s) clearly described?

Yes

Are the datasets clearly presented in a useable and accessible format?

Yes

Are the protocols appropriate and is the work technically sound?

Yes

Reviewer Expertise:

Coleoptera taxonomy

I confirm that I have read this submission and believe that I have an appropriate level of expertise to confirm that it is of an acceptable scientific standard.

# Reviewer report: Reviewer response for version 1

**DOI:** 10.21956/wellcomeopenres.26435.r121587

**Published:** 2025-04-07

**Authors:** Danilo Trabudo Amaral

**Affiliations:** 1 Universidade Federal do ABC (UFABC), São Paulo, Brazil

The manuscript presents a high-quality genome assembly of *Leptodirus hochenwartii*, offering a valuable contribution to the genomics of subterranean beetles. The rationale for creating the dataset is clearly described, highlighting the biological and historical relevance of the species and its underrepresentation in genomic databases. The methods and protocols are technically sound and align with current best practices in genome assembly and curation, including the use of PacBio HiFi reads, Hi-C scaffolding, and manual curation with established tools.

 Sufficient methodological details are provided to enable replication by other researchers, with links to protocols.io and open-access data repositories. The datasets are presented in a clear, accessible format, including genome assembly, annotations, and visualization tools such as BlobToolKit and Hi-C contact maps.

**Minor Considerations:**

- Consider replacing symbols like “~” with “approximately” in the narrative text for improved clarity and formality.
- Where appropriate, prefer active voice over passive constructions to enhance readability (e.g., “We assembled HiFi reads using Hifiasm...” instead of “HiFi reads were assembled...”).
- Review the manuscript for minor redundancies in phrasing; for example, “combined primary and alternate assemblies achieve...” could be simplified to “The combined assembly achieves...”.
- Define less common acronyms at first mention, such as “ToLID”, to ensure accessibility for a broader audience.
- Consider briefly discussing the biological significance of genome size and sex chromosome structure, as this could add context to the assembly’s relevance.
- A final sentence highlighting the potential taxonomic or evolutionary importance of this genome assembly would provide a more conclusive closure to the manuscript.

Are sufficient details of methods and materials provided to allow replication by others?

Yes

Is the rationale for creating the dataset(s) clearly described?

Yes

Are the datasets clearly presented in a useable and accessible format?

Yes

Are the protocols appropriate and is the work technically sound?

Yes

Reviewer Expertise:

Bioinformatics

I confirm that I have read this submission and believe that I have an appropriate level of expertise to confirm that it is of an acceptable scientific standard.
